# Primary care management of chronic insomnia: a qualitative analysis of the attitudes and experiences of Australian general practitioners

**DOI:** 10.1186/s12875-021-01510-z

**Published:** 2021-07-22

**Authors:** Jenny Haycock, Nicole Grivell, Anne Redman, Bandana Saini, Andrew Vakulin, Leon Lack, Nicole Lovato, Alexander Sweetman, Nicholas Zwar, Nigel Stocks, Oliver Frank, Sutapa Mukherjee, Robert Adams, R. Doug McEvoy, Elizabeth Hoon

**Affiliations:** 1 National Centre for Sleep Health Services Research, Adelaide, Australia; 2 FHMRI Sleep Health/Adelaide Institute for Sleep Health, College of Medicine and Public Health, Flinders University, Adelaide, Australia; 3 Sax Institute, Sydney, Australia; 4 Faculty of Medicine and Health, University of Sydney, Sydney, Australia; 5 College of Education, Psychology and Social Work, Flinders University, Adelaide, Australia; 6 Faculty of Health Sciences & Medicine, Bond University, Queensland Robina, Australia; 7 Discipline of General Practice, University of Adelaide, Adelaide, Australia; 8 Southern Adelaide Local Health Network, SA Health, Adelaide, Australia

**Keywords:** Insomnia, Sleep, Primary care, General practitioners, General practice, Family practice, Australia, Qualitative research

## Abstract

**Background:**

Chronic insomnia is a highly prevalent disorder, with ten to thirty percent of Australian adults reporting chronic difficulties falling asleep and/or staying asleep such that it causes significant daytime impairment. Current Australian general practice guidelines recommend cognitive behavioural therapy for insomnia (CBTi) as first line treatment for insomnia, however research suggests that most general practice consultations for insomnia result in a prescription for hypnotic or sedative medicines. Although the first point of contact for patients experiencing symptoms of insomnia is often general practice, little is known about the current role, experiences and capacity of Australian general practitioners to manage insomnia. This study aimed to address that gap by exploring the attitudes and opinions of general practitioners regarding insomnia management, to inform the development and implementation of new models of best practice insomnia care within general practice.

**Methods:**

A descriptive, pragmatic qualitative study. Purposive sampling was used to recruit practising Australian general practitioners, varying in age, years of experience and geographic location. Semi-structured interviews were conducted, and data analysed using thematic analysis.

**Results:**

Twenty-eight general practitioners participated in the study. Three major themes were identified: 1) Responsibility for insomnia care; 2) Complexities in managing insomnia; and 3) Navigating treatment pathways. Whilst general practitioners readily accepted responsibility for the management of insomnia, provision of care was often demanding and difficult within the funding and time constraints of general practice. Patients presenting with comorbid mental health conditions and insomnia, and decision-making regarding long-term use of benzodiazepines presented challenges for general practitioners. Whilst general practitioners confidently provided sleep hygiene education to patients, their knowledge and experience of CBTi, and access and understanding of specialised referral pathways for insomnia was limited.

**Conclusions:**

General practitioners report that whilst assessing and managing insomnia can be demanding, it is an integral part of general practice. Insomnia presents complexities for general practitioners. Greater clarity about funding options, targeted education about effective insomnia treatments, and referral pathways to specialist services, such as benzodiazepine withdrawal support and psychologists, would benefit insomnia management within general practice.

## Background

Chronic insomnia is a common sleep disorder, with 10–30% of adults in Australia reporting chronic difficulties falling asleep and/or staying asleep, to an extent that it causes significant daytime impairment [1–3]. Insomnia reduces work performance, social participation and health-related quality of life, and increases the risk of workplace and motor vehicle accidents [2, 4–6]. Chronic insomnia is associated with other chronic health conditions including cardiometabolic diseases [7, 8]. Comorbidity also exists between chronic insomnia and mental health conditions, most significantly depression and anxiety [9, 10]. Chronic insomnia clearly represents a major public health issue in Australia, supported by a 2019 Australian Government parliamentary inquiry which acknowledged the considerable impact of insomnia on Australian society, and recommended that sleep health become a national priority [11]. The serious risks associated with insomnia make access to effective and affordable treatments important.

In Australia, general practitioners (GPs), also known as family physicians, are central to primary care [12] and commonly provide treatment for sleep disorders. Most GPs in Australia work in privately-owned practices within a largely publicly funded health care system. GP consultations and the cost of most prescribed medications are subsidised by the Medicare Benefits Schedule (MBS) [13] and the Pharmaceutical Benefits Scheme (PBS) [14] respectively. In 2019–20, 87% of all GP consultations were provided free at point-of-care without any co-payment [15]. Funding streams exist within the MBS for the management of chronic diseases and mental health conditions [16, 17].

Guidelines published by the Royal Australian College of General Practitioners (RACGP) recommend cognitive behavioural therapy for insomnia (CBTi) as the first line treatment in the management of chronic insomnia, and advise that medication use should be limited to the lowest dose and the shortest duration possible [18]. Hypnotic or sedative medicines for insomnia are associated with high rates of adverse effects, and long term use can lead to dependence and issues with withdrawal [19–22]. Unlike medications, cognitive and behavioural modifications such as CBTi address the underlying psychological and behavioural factors that maintain insomnia and break the cycle of maladaptive learning, resulting in sustained improvements [23–26]. Whilst guidelines for insomnia management are available to Australian GPs, it is unclear if they are being utilised, with recent studies finding that Australian GPs rely heavily on sleep hygiene and pharmacotherapy when consulting with patients with insomnia rather than using cognitive behavioural treatments [20, 27, 28]. This represents a clear gap between recommended best practice management and current clinical care.

Previous research in this area, such as the work by Cheung et al. [27] and Sake et al. [28] recognises barriers to the integration of CBTi into the primary care setting in Australia, including an overreliance on pharmacotherapy, often based on perceived patient expectations, and inadequate support for non-pharmacological treatment pathways. These early studies recruited participants working in metropolitan areas in one state of Australia. The intention of this work was to further develop the knowledge in this area by purposely sampling GPs working in metropolitan, rural and remote locations Australia-wide. To understand key factors that influence the implementation of change we used a conceptual framework developed specifically for use in primary care [29].

Recently there has been growing interest in the development of new models of care for insomnia management in an attempt to improve usage and access to CBTi [30, 31]. This study was conducted to inform the development of new models of insomnia care by exploring the current role and experiences of GPs within insomnia assessment and management and by identifying barriers and facilitators that influence the provision of best practice insomnia care within general practice. This study was conducted as part of a larger research program exploring the implementation of new models of sleep health care within the primary care setting.

## Methods

### Study design

This qualitative study employed a pragmatic inductive approach within a larger mixed-methods program of research exploring the management of insomnia and obstructive sleep apnoea (OSA) in primary care. Semi-structured interviews were conducted and thematic analysis guided the data analysis [32]. Informed consent was obtained from all participants prior to participation in the study. Ethics approval was obtained from the University of Adelaide Human Research Ethics Committee (Reference H-2018-257).

### Setting

Participants were practising Australian GPs registered with the Australian Health Practitioner Regulation Agency. Participants practising in metropolitan, rural and remote regions of Australia were included as it was thought that participant’s experience in insomnia management could vary with distance from metropolitan specialist services.

### Recruitment strategy

Purposive sampling was undertaken through two recruitment sources, a GP-led closed Facebook group GPs Down Under (https://www.facebook.com/groups/gpsdownunder/about) and professional networks of the research team, using advertising that explicitly stated that the study was about improving care for patients with sleeping issues. This sampling strategy aimed to recruit an information-rich sample [33] varied in terms of age, level of experience, rurality and state of Australia. Recruitment continued until data saturation was achieved, whereby new interview data provided few further insights (and no new themes) beyond data previously collected [34].

### Data collection

Data were collected between February and August 2019. A semi-structured interview guide was developed, informed by both clinical experience of the researchers and a conceptual framework by Lau et al. [29] that identifies professional, organisational and external factors that influence change within primary care. A multi-disciplinary group of insomnia and OSA specialists, academic GP clinician/researchers, sleep researchers and qualitative experts met to contribute to the development of two interview guide drafts, one with fewer and more open questions than the other. Iterations continued until consensus was reached on the two versions. The guides were then piloted with three GPs, which provided insight into further refinement and ultimately the development of the final interview schedule, which combined the most effective parts of both versions, that was used within this study. As there were limited changes to the interview guide post-piloting, these data were included in the study. Except for one face-to-face interview at the request of the participant, all interviews were conducted by telephone by an experienced qualitative researcher (EH) and a primary care registered nurse/research assistant (NG). Interviews were audio recorded with consent of participants and transcribed verbatim. Reimbursement ($150 AUD) for participation was provided, commensurate with an accepted hourly rate for GPs.

### Data analysis

An inductive thematic analysis was undertaken to analyse the data [32]. Three researchers (EH, NG and JH) familiarised themselves with the data individually and all transcripts were checked for accuracy. Data were coded independently with NVivo 12 software (QSR International Pty Ltd, Doncaster, Victoria, Australia) using an open coding method with each of the researchers developing initial codes, followed by multiple discussions until reaching an agreed coding framework. All three researchers identified patterns and relationships in the data and developed potential themes through the process of thematic analysis [32]. These themes were then discussed and refined by the three researchers until reaching a consensus on the final themes reported within this paper.

## Results

### Participant characteristics

A total of 28 GPs participated in the study. Participants practised as a GP across the six states of Australia, with 61% (*n* = 17) working in a metropolitan area, 32% (*n* = 9) in a rural area and a further 2 participants (7%) in a remote location. Years working as a GP varied greatly within the group, ranging from 0.3–42 years (median 6 years). Most participants reported working part time, with only four participants (14%) working full-time (mean 0.6 [range 0.2–1.0] full-time equivalent). Sixty-one percent of participants identified as female (*n* = 17). Seventy one percent of participants reported consulting with at least 5 patients with insomnia each month. More detailed participant characteristics are presented in Table 1.

**Table 1:** Participant characteristics

**Participant**	**Rurality**	**Age group**	**Gender**	**Years practising as a GP**
P1	Rural	25–34	Female	3.0
P2	Rural	35–44	Male	4.0
P3	Metropolitan	25–34	Male	5.0
P4	Metropolitan	25–34	Female	2.0
P5	Rural	25–34	Female	3.0
P6	Metropolitan	35–44	Male	1.0
P7	Metropolitan	55–64	Female	29.0
P8	Metropolitan	35–44	Female	6.0
P9	Metropolitan	55–64	Female	26.0
P10	Metropolitan	35–44	Female	6.0
P11	Metropolitan	55–64	Male	35.0
P12	Metropolitan	55–64	Male	42.0
P13	Metropolitan	35–44	Male	10.0
P14	Rural	25–34	Female	5.0
P15	Metropolitan	45–54	Female	20.0
P16	Metropolitan	35–44	Female	17.0
P17	Metropolitan	35–44	Female	0.3
P18	Metropolitan	35–44	Female	3.0
P19	Metropolitan	35–44	Female	14.0
P20	Rural	55–64	Male	30.0
P21	Remote	45–54	Male	20.0
P22	Rural	55–64	Male	30.0
P23	Rural	55–64	Female	30.0
P24	Rural	25–34	Male	4.0
P25	Remote	25–34	Male	5.0
P26	Metropolitan	25–34	Female	5.0
P27	Metropolitan	45–54	Female	26.0
P28	Rural	25–34	Female	2.0

Following analysis of the data, three major themes were identified:

1. Responsibility for insomnia care
2. Complexities in managing insomnia
3. Navigating treatment pathways

### Theme 1: responsibility for insomnia care

This theme identified a sense of responsibility for insomnia management by GPs. Participants consistently recognised that the management of insomnia was within the scope of general practice.

*For insomnia, I probably don’t see that as a referral out of general practice very often. Unless there’s a very significant mental health component to it. (Participant 7)*

*I can’t remember ever referring someone for insomnia. Maybe once I’ve referred someone to a psychologist, but not – generally, no. That’s something that to me sits in the scope of general practice. (Participant 22)*

A number of participants stated that they would initiate referrals to other services as needed, but this was most commonly for the management of co-morbid insomnia and another condition such as anxiety or depression, or for cases of severe, chronic insomnia.

*It depends on the severity and depends on if there’s…a comorb with psychiatric illness then I would probably be referring on to my psychiatric colleagues. (Participant 5)*

*I think when it gets to that sort of [chronic] level I would probably outsource it, so I would probably speak to a sleep clinic or a sleep psychologist, if that were the case. (Participant 8)*

Although the management of care for patients with insomnia was considered routine practice by participants, the time taken to provide the care was not so readily accepted. Many GPs reported concerns about the significant demands that insomnia management entailed.

*If…someone were to come in for a 10 min appointment and go through insomnia, that would take about 15 to 20 min, and the pressure of running late, keeping someone else waiting tends to start playing on you at some point down the line. (Participant 10)*

*Teasing out the things that are contributing to it takes a lot of time, and then if you’re wanting to change particular behaviours and then doing the motivational interviewing to go with that then that takes time. (Participant 18)*

Participants reported that for most patients, insomnia symptoms presented with symptoms of other co-morbid conditions, and that unpacking the issues relating to insomnia took time and contributed to longer consultation times.

*In terms of time limitations, it’s often brought up in the context of multi-disciplinary comorbid care, so it takes a long time to sort these things out and trying to disentangle it from all the multiple presentations, and patients very rarely present with insomnia as their sole issue. (Participant 7)*

Funding constraints were identified as limiting more comprehensive insomnia management. Many GPs reported that the current MBS funding model incentivised shorter consultation times, resulting in those offering longer appointments being disadvantaged financially.

*You’re kind of taking on a time bomb [providing insomnia management], because…if you can put through three people every 15 min, you’re going to get paid a lot more than those really long extended consults. So while it’s rewarding, it’s not financially rewarding. That’s the sticking point. (Participant 16)*

One participant stated that, although he was interested in being more involved in the management of insomnia, he was currently unwilling to provide more comprehensive care due to the funding limitations associated with the extended time required to provide insomnia management.

*Unless there was…a stellar rebate for it…I’m not doing that on a – on the Medicare, what, 36 bucks or whatever they pay us. (Participant 6)*

### Theme 2: complexities in managing insomnia

This theme highlights the greatest challenges presented to GPs’ managing patients with insomnia, namely overlapping insomnia and mental health conditions, and the management of patients taking benzodiazepines.

Many GPs reported that consultations for the management of insomnia were complex. Participants reported that care of insomnia was rarely straightforward, with patients commonly presenting with insomnia symptoms in the presence of another comorbid condition, and that the care of insomnia could be demanding.

*Often they’re hard…patients [to see] as well, because there’s often a lot of other complex issues going on as well. It’s not just [that they] can’t sleep. (Participant 26)*

Insomnia was also not always a priority for patients, with patients often presenting for care of the other conditions comorbid with insomnia rather than for management of the insomnia itself.

*It’s normally, “And by the way, I’m having trouble sleeping. I’ve come in for this, but by the way…” So it tends to be…the bigger of the two issues, but they don’t perceive it as that. (Participant 10)*

Participants recognised a strong correlation between mental health and insomnia. Most commonly, GPs related a complex comorbidity between insomnia, depression and anxiety. Some GPs reported that at times, it was only through an assessment for mental health issues that insomnia symptoms were identified.

*Some, if they are depressed, part of their screening is you ask about their sleep and things, when they are anxious…if there is any mental health complaint as part of the screening, you could ask about sleep, and you realise it’s a lot of that. (Participant 1)*

Some participants reported that mental health issues and insomnia were often so interrelated that it is necessary to treat them concurrently.

*If they keep coming in – if it’s due to an underlying depression, then…you go, “How’s the sleep? And how’s your mood?” So it’s very much interrelated. You can’t sort of separate it. (Participant 10)*

A distinction between insomnia and mental health was not always clear, with one participant stating that it may be difficult for some GPs to recognise insomnia in the midst of mental health symptoms.

*A lot of doctors ended up taking, you know, the fatigue, the depression, but that’s not the real cause of the problem. That’s a consequence of the chronic insomnia. (Participant 1)*

Of note, one GP considered insomnia not as a condition in itself, but rather as a symptom of other conditions.

*Insomnia isn’t actually a disease. It’s a symptom only…insomnia doesn’t happen by itself. (Participant 11)*

Benzodiazepines also presented complexities for GPs. Many participants recognised risks associated with long-term use of benzodiazepines and were reluctant to prescribe benzodiazepines beyond the short term.

*I really, really try to avoid it [prescribing benzodiazepines]. Just knowing the harm that it can potentially do, and I just feel like it’s quite a Band-Aid. It doesn’t solve the issue at hand. It’s not a long-term solution. Yes. And I certainly make that very clear up front with my patients these days. (Participant 14)*

Some participants, whilst reluctant to prescribe benzodiazepines, acknowledged that there were some situations in which they were required.

*Even though sometimes you can’t find – even if you find the reason, you still end up having to use some sort of a chemical medication like a benzodiazepine sometimes. (Participant 11)*

GPs frequently reported that they prescribed benzodiazepines in very limited amounts in acute situations or for shift-work disorder.

*I very, very, very rarely use benzodiazepines. I think basically, my use for benzodiazepines would be restricted to a grief type scenario…then I might actually just give them three tablets or something. I’m quite a miser with such things. (Participant 8)*

*With shift work insomnia, I may use a benzodiazepine, and I give them at the start of a – you know, when they finish their nightshift, and they’re trying to get their sleep/wake cycle back to normal. (Participant 21)*

Contributing to the GPs’ reluctance to prescribe benzodiazepines was an understanding of the problematic consequences of long-term use. Many participants described a group of patients that had long-term dependence on benzodiazepines, often ‘inherited’ from other GPs, from times when the awareness of risks of benzodiazepines was less apparent.

*And 20 or 30 years ago the practice of using benzos was obviously a lot more prevalent, and so we’ve inherited people who have been on their Temazepam or their Serepax for 30 years, and you try to get them off of it, but it just doesn’t work, because they’re so dependent. (Participant 16)*

Denying a prescription of benzodiazepines to these patients was challenging and many GPs reported difficulties in achieving treatment change with these patients. Maintaining rapport with patients, whilst attempting to wean them off benzodiazepines, was difficult, particularly when the GP was not well known to the patient.

*Well, back in the sixties, everyone got that, so everyone was addicted, and so you have a lot of people in their seventies…[that] have been on things like that for 20 years, and you are a brave person to try and change the direction the wind blows. (Participant 13)*

*So I just have to try and establish some trust and rapport with them, so that they can trust what I’m saying…There has been one doctor that prescribed for maybe a long time, and then maybe that doctor has retired or they will see someone else for whatever reason, and then…suddenly, they find they’ve got trouble getting the medication…the patient feels like it’s their fault that they’ve become addicted to the medication or that they’re seeking it when…in their mind, a doctor prescribed it to treat their insomnia, so the patients will get a little bit defensive as well. (Participant 4)*

For some GPs the challenges of supporting long-term patients to curtail their use of benzodiazepines was so difficult that it had led to a termination of their relationship, with patients instead seeking ongoing prescriptions elsewhere.

*So if I don’t give them what they want, after my 22 min in a 15 min booking, they will go to the 6 min medicine man around the corner and get what they want. (Participant 15)*

*I just feel like I have to continue, but I will only agree to be their doctor – part of the deal is we try and wean [benzodiazepines] down. So – have had some – you lose a bunch of patients, because of that. (Participant 6)*

For one GP, the challenge of withdrawing benzodiazepines resulted them being placed in a life-threatening situation.

*But with benzos, we’ve definitely had…threatened violence, threatened use of weapons…losing a litre of blood, because they didn’t get the medications they’re after. So we’re in a very confronting kind of situation. (Participant 27)*

The difficulties of managing patients seeking long-term benzodiazepine prescriptions resulted in GPs seeking alternative care options for their patients. Several participants stated that they referred patients to psychiatrists for review of their benzodiazepine use once they had exhausted all approaches available to them.

*I referred a few people to the psychiatrists, because we’ve had – like they’re wanting long term prescription for benzodiazepines, and I felt that I reached the end of what I could offer. (Participant 4)*

Other participants reported a need for specialist addiction support but identified a lack of appropriate services available to assist these patients with benzodiazepine withdrawal.

*And then for all those troublesome patients who are in their 70 s and who are now on two tablets of Temazepam and have been for 50 years maybe there is somewhere that I can refer them to do this in a joint [way] between the specialists and I, and go, right, let’s get this down. That would be useful. (Participant 10)*

*If there was a service available to help with benzodiazepine, like addiction and withdrawing slowly, that would be excellent…even if the patient didn’t want to engage with them…If there was a decision-making tool that could help with making a withdrawal plan that would be excellent. (Participant 4)*

### Theme 3: navigating treatment pathways

This theme reports the approaches used by GPs when providing care for patients with insomnia. It is apparent from this theme that, whilst GPs consistently provided sleep hygiene, their approach to managing complex insomnia was more varied.

The provision of sleep hygiene, that is habits that are considered to be conducive to good sleep such a reducing caffeine intake and regular bedtimes, was commonly reported by participants as a strategy used to manage insomnia. Almost all of the GPs interviewed expressed confidence in providing advice about sleep hygiene with most stating that it was their first line of treatment for patients presenting with symptoms of insomnia.

*Sleep hygiene I recommend to everyone. So I’ve got some handouts and stuff for sleep hygiene that I give out to people if it’s clear that their sleep hygiene is poor. (Participant 21)*

For some GPs offering sleep hygiene education was used as a means to avoid prescribing benzodiazepines, and for others, a way of supporting patients looking for a ‘quick fix’ for their insomnia. One GP reported that this ‘simple’ education was, at times, all that was needed to treat insomnia.

*There’s this expectation that you’re going to fix and clearly insomnia is not something that you’re going to fix today, but having something to give to patients as like a takeaway pack of written down instructions of sleep hygiene, that would be really helpful. You would feel like you’ve given them something, and it’s almost like a deflector for the requests for benzos. (Participant 16)*

*Sometimes we give advice that, to us, seems ridiculously simple and the patient comes back and says they’re totally cured, and it’s all wonderful, and we feel we haven’t done much. (Participant 12)*

However, many participants acknowledged that insomnia management was not always straightforward. The approach to managing more complex cases of insomnia by GPs varied, as did their knowledge about insomnia management strategies and referral pathways. Some GPs reported an awareness of cognitive behavioural therapy (CBT) as one element of managing insomnia, particularly the use of CBT to target anxiety associated with insomnia.

*Often there’s an element of anxiety…so you run them through mindfulness and a bit of CBT and meditation and that sort of stuff to help them chill out a bit and relax. (Participant 25)*

Only three participants referred to cognitive behavioural therapy for insomnia (CBTi) within the interview and only after the term was introduced by the interviewer, and very few were aware of specific CBT techniques for insomnia. Three GPs reported offering a variation of sleep restriction therapy, however there was no acknowledgement by those participants of it being a component of CBTi.

*Sometimes I would do something like sleep restriction with them, getting them to work out how many hours of sleep they’re actually getting and then delaying bedtime until that and bringing it back however many stepwise. (Participant 8)*

Some GPs expressed uncertainty about referral options for complex cases of insomnia. As reported in Theme One, participants referred patients to other health professionals when required, but at times they were unsure about the referral pathways available to them. Some GPs were comfortable referring patients to a psychologist or psychiatrist, but others were unsure whether it was appropriate to refer a patient to a sleep physician. Reasons for this varied, including a lack of understanding of the role that sleep physicians play in insomnia management and a belief that insomnia may not be significant enough for a referral to a specialist.

*Probably consider a sleep physician, but then again, I don’t know. It depends what I feel like they might be able to offer. (Participant 14)*

*I wouldn’t necessarily refer someone to a sleep physician for this. I don’t even know if they – now, that I’m talking to you, maybe they do it, maybe they don’t. I actually have no idea. (Participant 10)*

*If I’m not getting anywhere with all of that, I guess, referring for specialist in – but I would very, very rarely refer to a sleep specialist just for insomnia. (Participant 28)*

When referral pathways were available, cost was identified as a barrier to treatment, with one GP reporting that it was cheaper for patients to pay for medication than to see a psychologist.

*I would say that’s probably like less than five per cent of my patients would actually go to someone to talk about their sleep issues, because of the cost, the cost limiting factor. (Participant 15)*

*It’s much easier to pay $6.90 for Temazepam than going to see a psychologist for my sleep. (Participant 4)*

There was also uncertainly expressed about the appropriateness of using an MBS funded GP Mental Health Treatment Plan (GPMHP) for the management of insomnia. Despite distress from sleep issues being an eligible condition for a GPMHP [35, 36], insomnia was almost never considered by GPs as the primary diagnosis for a GPMHP and several participants believed that insomnia was not an eligible condition for a GPMHP.

*On the mental health plan that I am sending with them, the diagnosis is probably not primarily insomnia, because that doesn’t fit within the guidelines for mental health care plans. (Participant 23)*

Of the participants who reported creating a GPMHP in the context of insomnia management, almost all considered insomnia as a secondary condition for the plan, co-existing with a mental health condition such as anxiety and/or depression, that was used as the primary diagnosis for the GPMHP.

*I’ve referred someone for mental health issues that were contributing to insomnia, but that’s a referral not for the insomnia, that’s for the mental health condition. (Participant 22)*

*I think I’ve probably got a lot of patients – well, a reasonable number of patients who have insomnia who are on a mental health care plan for their depression or their anxiety or anything like that, but purely – I don’t think I have put them on for insomnia. (Participant 24)*

## Discussion

This qualitative study provides insights into barriers or facilitators that could influence the implementation of new models of insomnia care within general practice, specifically those that had the potential to increase the delivery of evidenced-based behavioural treatments as an alternative to medications.

The findings of this study indicate a discrepancy between current clinical practice and the RACGP clinical guidelines which recommend CBTi as the first line treatment for insomnia [18]. GPs rely on sleep hygiene education treatment with scarce reference to the other components of CBTi, consistent with previous research on this topic [37–40]. This is of concern given limited evidence about the effectiveness of sleep hygiene as a standalone treatment in primary care [41]. Education for GPs targeting efficacy of insomnia treatments should be considered with the aim of improving uptake of CBTi, the most effective treatment.

A finding within this study that has not been explored in previous work on this topic is the considerable difficulties experienced by GPs managing patients that they have inherited from other GPs who have been prescribed benzodiazepines for extended periods of time. Previous research indicated that the majority of insomnia patients in primary care are managed with pharmacotherapy [20], whereas GPs within this study consistently reported that they avoid benzodiazepines as the first-line, or long-term treatment of insomnia*.* These GPs highlighted specific challenges involved in managing patients who had been taking benzodiazepines for many years: difficulties maintaining relationships with patient’s seeking benzodiazepine prescriptions, and a lack of accessible support services targeting medication withdrawal. It is of note that while in this study benzodiazepines were specifically identified by participants as complex to manage, all medications prescribed for insomnia have associated challenges, such as side effects and issues of dependency [42]. Health professionals not only need education and training on benzodiazepine withdrawal, but also require increased accessibility of alternative management options to drug treatment and medication withdrawal support services [43].

This study highlighted that insomnia presentations are complex and often overlap with mental health conditions. The Better Access initiative in Australia [17] provides access to mental health trained professionals through use of a GPMHP, but a novel finding of this study was that most GPs did not recognise insomnia as a diagnosis appropriate for a GPMHP. Insomnia was sometimes mis-conceptualised as a secondary condition by participants despite changes to diagnostic criteria in 2005 which recommended that ‘comorbid insomnia’ instead be used to encourage targeted diagnosis and treatment of insomnia per se [5]. This finding again highlights the need for education and training for GPs, focussed on diagnostic criteria for insomnia and use of GPMHPs for chronic insomnia.

Maintaining a clinical relationship with patients, developing trust and rapport, and managing patient expectations were identified as important in managing this complex condition, but also very demanding and time-consuming within a primary care consultation. This is of particular importance given that other studies have identified that maintaining patient satisfaction and good relationships with patients can be drivers for GPs prescribing benzodiazepines for insomnia [38]. This study also confirmed [27] how the current Medicare funding structure favouring financial reimbursement to the GP for short, timed consultations, makes longer and comprehensive treatment for complex conditions such as insomnia, challenging. CBTi, the recommended first-line treatment for insomnia is generally administered over weekly 30–60 min sessions, which may be difficult to achieve in primary care. Potential solutions could include improved financial incentives for longer consultations, task shifting to practice nurses/practice pharmacists and education about the use of GPMHPs for insomnia management [16, 17]. More condensed brief behavioural therapy for insomnia (BBTi) could also be considered for use in Australian general practice [44–46].

In line with other recent research [27, 28], GPs’ management of insomnia is also influenced by a lack of viable referral pathways to specialist care for complex cases. Awareness of professional networks is important to provide the most effective care to patients with complex insomnia. For some GPs there are no viable pathways because of accessibility issues related to cost and geographic distance from appropriate services. GPs were unsure whether referral to a sleep specialist was appropriate for insomnia, possibly reflecting an understanding that sleep specialists are almost exclusively sleep apnoea specialists. Local Health Pathways [47] are being developed in various Primary Health Networks across Australia, and where these primary care focussed systems are able to identify accessible local referral pathways, they could potentially be useful tools for Australian GPs when providing care for people with insomnia.

To improve care for people with insomnia, other modalities and treatment options for CBTi should be considered. Stepped care models where incremental levels (steps) of intervention complexity have proven to be effective in the treatment of insomnia [31] and could be evaluated in general practice. Many GPs provide sleep hygiene education, and this information could be upgraded to include self-help CBTi treatments [48]. Several online CBTi treatment programs are now available and have the potential to be further utilised in general practice [49].

This research has provided a broader understanding of the context of insomnia management within general practice in Australia. Expanding on implementation work conducted by Koffel and Hagedorn [50], a recommendation is that this research be used to inform implementation of new models of care for insomnia management in the primary care setting. The Adelaide-based National Centre for Sleep Health Services Research (https://www.ncshsr.com) has drawn on the study’s findings to design implementation trials of new technologies and clinical pathways to increase GP and patient access to guideline-recommended CBTi. It will use these and other research findings as it endeavours to bring about Australia-wide clinical change in the primary care management of insomnia [30].

### Study strengths and limitations

The use of appropriate research strategies and techniques was a key strength of this qualitative study and ensured rigour throughout the study design, sample selection, data gathering and analysis phases [51]. These strategies included purposeful sampling to allow for a maximally varied sample, consistent data collection techniques until data saturation was reached, and ongoing analysis by multiple researchers enabling triangulation. The perspectives of participants were emphasised with thick description by the inductive approach to data collection and analysis [52]. A limitation of this study was that an understanding of the term insomnia was not explored with participants, with it unclear at times if GPs were reflecting on their experiences managing chronic insomnia or sleep difficulties more broadly.

## Conclusions

It is evident from this study that GPs recognise that the management of insomnia is within the scope of general practice, but that managing this condition can be difficult. Chronic insomnia is often a complex health problem, frequently associated with co-morbid mental health conditions and benzodiazepine dependency. Whilst this study demonstrates that there are some barriers to change in the management of insomnia in primary care, there are also many facilitators supporting the implementation of new models of care. By harnessing the motivation and interest of GPs to be involved in insomnia management, there would appear to be some specific opportunities to improve the provision of care for patients presenting with insomnia to the general practice setting within Australia. By providing GPs with relevant and targeted education and resources, including appropriate financial reimbursement through the MBS system and support for managing benzodiazepine withdrawal, GPs may be empowered to better address the needs of the many patients living with chronic insomnia.

## Data Availability

The datasets generated and/or analysed during the current study are not publicly available due to concerns participant privacy may be compromised but are available from the corresponding author on reasonable request.

## Abbreviations

BBTi: Brief behavioural therapy for insomnia
CBT: Cognitive behavioural therapy
CBTi: Cognitive behavioural therapy for insomnia
DSM-V: Diagnostic and Statistical Manual of Mental Disorders
GPs: General practitioners
GPMHP: General Practitioner Mental Health Treatment Plan
MBS: Medicare Benefits Schedule
OSA: Obstructive sleep apnoea
PBS: Pharmaceutical Benefits Scheme
RACGP: Royal Australian College of General Practitioners
